# Gender and activity disparities in the relationship between circadian syndrome and gallstone disease

**DOI:** 10.3389/fendo.2024.1439514

**Published:** 2024-12-23

**Authors:** Binbin Feng, Tianlin Wang, Junquan Han, Zhaoshuai Yang, Hong Wang, Huizhen Li

**Affiliations:** ^1^ Graduate School, Tianjin University of Traditional Chinese Medicine, Tianjin, China; ^2^ Department of Gastroenterology, Tianjin University of Traditional Chinese Medicine Second Affiliated Hospital, Tianjin, China; ^3^ Department of General Surgery, Tianjin University of Traditional Chinese Medicine Second Affiliated Hospital, Tianjin, China

**Keywords:** gallstone disease, circadian syndrome, metabolic syndrome, cross-sectional study, National Health and Nutrition Examination Survey (NHANES)

## Abstract

**Objective:**

To explore the potential correlation between gallstone disease (GSD) prevalence and circadian syndrome (CircS).

**Methods:**

The cross-sectional research utilized data spanning 2017 to March 2020, sourced from the National Health and Nutrition Examination Survey (NHANES). The GSD data were collected via questionnaires, with appropriate sample weights applied to ensure the study population was representative. Three multivariable logistic regression models were built to clarify the connection between CircS and GSD. Furthermore, subgroup analysis and interaction test were carried out, categorized based on demographic traits and lifestyle aspects, to discern the potential influence of these variables on the correlation.

**Results:**

The analysis included 4,126 participants, with a prevalence of 38.68% for CircS and 12.04% for GSD. The multivariable logistic regression analysis indicated a positive correlation between CircS and the prevalence of GSD (Odds Ratio (OR) = 1.336, 95% Confidence Interval (CI): 1.048, 1.702). When stratified by the number of CircS components, a positive correlation was observed between the number of CircS components and the prevalence of GSD (P for trend < 0.05). In particular, individuals with six or more CircS components had a higher prevalence of GSD than those with three or fewer components (OR = 2.608, 95% CI: 1.464, 4.647). The subgroup analysis and interaction test revealed that a positive correlation between CircS and GSD prevalence was mainly observed in female individuals (OR = 1.701, 95% CI: 1.236, 2.341) and individuals not engaged in moderate activity (OR = 1.990, 95% CI: 1.158, 3.418).

**Conclusion:**

There is a positive correlation between CircS and GSD prevalence, particularly among females and individuals not engaging in moderate activity. These findings offer new insights for research directions in GSD and may impact preventive and therapeutic strategies.

## Introduction

Globally prevalent, gallstone disease (GSD) significantly contributes to hospital admissions for gastrointestinal disease (1, 2). Across Europe, the United States, and other developed countries, the occurrence rate of GSD varies between roughly 10-15% in adults (3, 4). Globally, the occurrence of GSD varies significantly among various ethnicities. For example, the occurrence rate reaches up to 70% in American Indians and ranges between 10% and 15% in white adults (5, 6). Conversely, the occurrence rate is comparatively lower among Asian groups (7). While GSD usually shows no symptoms, about 10% to 25% of patients might suffer from conditions like biliary pain and acute cholecystitis (8). Severe complications can occur in 1% to 2% of patients, increasing the likelihood of conditions such as pancreatitis, gallbladder cancer, and liver cancer (9–11). It’s estimated that averting and managing GSD in the U.S. incurs an annual economic cost of around $62 billion, imposing significant monetary pressure on healthcare infrastructures (12). Due to these factors, GSD is acknowledged as an essential concern in public health. Despite previous research identifying various risk factors for gallstone development, including genetics, environmental influences, dietary habits, and individual metabolic status, reliable clinical indicators for the prevention of GSD remain scarce (6, 8).

The circadian rhythms, controlled by a brain’s internal clock with a central pacemaker and external organ clocks, govern the sleep-wake cycle and various other bodily functions, significantly influencing human health and metabolic processes (13, 14). Unhealthy living habits, including sleep disturbances, artificial light exposure, and shift work, may interfere with these rhythms, resulting in negative health consequences. Numerous studies have demonstrated the association between circadian rhythm disruption and metabolic syndrome (MetS), including its core components such as abdominal obesity, hypertension, hyperglycemia, and dyslipidemia (15–21). Moreover, this disruption is also associated with major comorbidities of MetS, including sleep disorders and depression (22–24). Consequently, some researchers suggested circadian rhythm disruption could be a significant underlying factor contributing to MetS (21, 25). This has prompted the development of the novel concept of circadian syndrome (CircS), which is intricately linked to circadian rhythms (21). Diagnosing CircS involves identifying at least four chronic ailments: high blood pressure, abnormal lipid levels, enlarged waist size, diabetes, reduced sleep time, and depression (26). Research has confirmed a close correlation between MetS and its core components with GSD. A cross-sectional study involving 7,570 participants, including 918 patients with gallstones and 6,652 healthy controls without gallstones, confirmed the close correlation between GSD and MetS and found that the more components of MetS, the higher the prevalence of GSD (27). In another study, researchers compared 200 patients with GSD post-laparoscopic cholecystectomy to 200 contemporaneous controls without gallstones, revealing a close correlation between the prevalence of GSD and MetS (28). The study also identified hyperglycemia and low levels of high-density lipoprotein cholesterol (HDL-C) as independent risk factors for GSD (28). Mendelian randomization studies indicated an increased risk of GSD in patients with MetS, particularly in those with abdominal obesity (29). Meta-analyses provided additional evidence of an elevated risk of developing MetS in individuals with GSD (30). Furthermore, sleep issues and depression, frequently accompanying MetS, are acknowledged as contributing factors to the development of GSD. Animal experiments have shown that circadian rhythm disorders can promote the formation of gallstones in mice and revealed that this may be related to abnormalities in bile acid and cholesterol metabolism in the mouse liver (31). A retrospective cohort study involving 18,141 Chinese elderly individuals revealed that patients with depression have a higher risk of developing GSD, and further Mendelian randomization studies have suggested that abdominal obesity may mediate this association (32). Additionally, a cross-sectional study based on the National Health and Nutrition Examination Survey (NHANES) database also found that depression patients have an increased risk of gallstone prevalence, and Mendelian randomization studies have further confirmed the causal relationship between them (33). Consequently, investigating the possible link between CircS and GSD prevalence among American adults is crucial.

As far as we are aware, there have been no investigations into the relationship between CircS and the prevalence of GSD. Consequently, this cross-sectional study aims to explore the connection by conducting a thorough analysis based on the NHANES data and determine whether sociodemographic factors (age, gender, race, education level, marital status) and lifestyle factors (smoking status, alcohol status, vigorous activity, and moderate activity) affect this correlation.

## Materials and methods

### Study population

**Figure 1:**
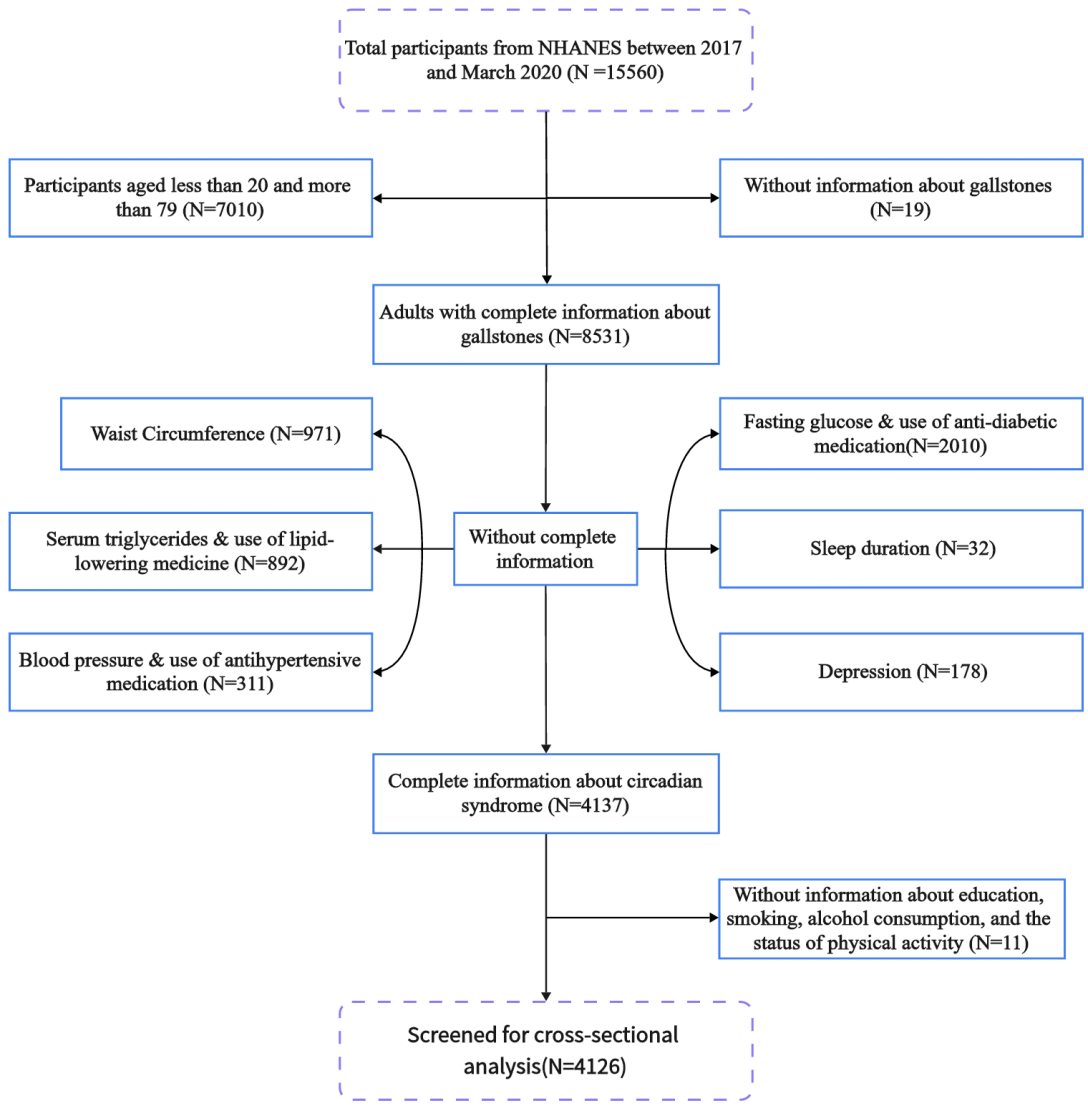
Flowchart of study population inclusion in the 2017–2020 NHANES. NHANES, National Health and Nutrition Examination Survey.

The data for this research were sourced from NHANES, covering the period from 2017 to 2020. Biennially, NHANES carried out a sequence of multi-stage surveys, executed by the Centers for Disease Control and Prevention. Field activities were paused in March 2020 due to the COVID-19 pandemic, resulting in incomplete data collection for the 2019-2020 period. Addressing this problem, NHANES amalgamated data gathered between 2019 and March 2020 and 2017 to 2018, creating a sample representative of the national pre-pandemic population from 2017 to March 2020. Initially, our study included 15,560 participants. After excluding individuals under 20 and over 79 years of age, totaling 7,010 individuals, and an additional 4,424 participants with incomplete demographic, physical, and disease information, the final analysis included 4,126 participants (details in **Figure 1**). Protocols for the NHANES study received approval from the Ethics Review Board of the National Center for Health Statistics Research, with every participant providing informed consent (details can be found at https://www.cdc.gov/nchs/nhanes/irba98.htm).

### Diagnosis of gallstones

The main outcome of this research was the GSD prevalence. Diagnosing GSD relied on the NHANES medical condition questionnaire. Trained researchers conducted these surveys via face-to-face interviews. Identifying GSD was based on how participants responded to the question “Has a doctor or other health professional ever informed you about having gallstones?” Those who confirmed this diagnosis were diagnosed with GSD.

### Diagnosis of circadian syndrome

Circadian syndrome was identified as the key variable for exposure, with its diagnosis hinging on these factors: individuals who met four or more of these criteria were considered to have this syndrome (26). Identified criteria included: central obesity, characterized by a waist size exceeding 102 cm in men and 88 cm in women; high blood triglyceride concentrations (≥150 mg/dL) or taking lipid-lowering drugs; reduced HDL-C levels (<40 mg/dL in men, <50 mg/dL in women) or taking lipid-reducing drugs; increased blood pressure (systolic ≥130 mmHg or diastolic ≥85 mmHg) or taking blood pressure-lowering drugs; high fasting blood sugar levels (≥100 mg/dL) or taking medication for low blood sugar; insufficient sleep time (≤6 hours daily); and depression, as evaluated by the NHANES mental health study (26, 34–36). People who achieved a score of 10 or above in the Patient Health Questionnaire-9 (PHQ-9) survey were identified as suffering from depression (37).

### Covariates

This research took into account possible confounding variables. Age was factored into continuous variables, whereas categorical variables encompassed gender, ethnicity, marital status, educational attainment, smoking habits, alcohol intake, vigorous activity, and moderate activity, with age being categorized accordingly. The specified age groups were: 20-34 years, 35-49 years, 50-64 years, and 65-79 years (36, 38). Racial classifications included Mexican Americans, other Hispanics, non-Hispanic whites, non-Hispanic blacks, or other ethnicities (39). The classification of marital status encompassed married/living with the partner, widowed/divorced/separated, or never married (40). The categorization of educational attainment was segmented into less than high school, high school, and more than high school (41). The status of smoking (SMQ020) and alcohol (ALQ111) were ascertained using the respective survey (42–44).

### Statistical analysis

To ensure the representativeness of our results across the entire US population, statistical evaluations were performed using the relevant sampling weights from the NHANES. To characterize the participants’ traits, continuous variables were analyzed through survey-weighted linear regression, presenting the results as weighted averages and 95% confidence intervals (CI). The analysis of categorical variables was conducted using a survey-weighted chi-square test, with results presented as weighted percentages along with 95% CI.

To investigate the link between CircS and GSD, participants were classified based on their CircS diagnosis, and three weighted multivariable logistic regression models were created for evaluation. Furthermore, an evaluation was conducted on the relationship between the quantity of CircS components and GSD. Within the crude model, no variables underwent adjustments. Adjustments were made in Model I to account for variables such as age, gender, and ethnicity. Expanding on Model I, Model II incorporated additional modifications considering factors such as educational level, marital status, smoking status, alcohol status, vigorous activity, and moderate activity. For trend analysis, the number of CircS components was considered as a continuous variable. Additionally, to investigate if covariates influence the link between CircS and GSD, we performed subgroup analysis and interaction test for all potential confounding variables.

A P-value below 0.05 was deemed statistically significant. Every statistical analysis was performed using EmpowerStats (version 4.2) and the R software (version 4.3.2, R Foundation, Vienna, Austria).

## Results

### Baseline characteristics of the population

**Table 1** displays the traits of the study subjects, categorized according to the Circs status. The study eventually encompassed 4,126 participants, following a selection procedure that removed those not fitting the age criteria and those with incomplete information. After applying the sample weights, the average age of the participants was 48.93 years, and the prevalence rate of Circs was 38.68%. In addition to gender, race, alcohol status, and activity status, the two groups showed significant differences in baseline characteristics (P< 0.05). Individuals with CircS were more prone to GSD, generally had older age, lower educational attainment, smoking habits, and were less frequently never married.

**Table 1:** Characteristics of participants by categories of circadian syndrome status, weighted.

Variables	All (*N*=4126)	Groups		*P*-value
		Non-circadian syndrome	circadian syndrome	
		(*N*=2323)	(*N*=1803)	
Age(years)	48.93 (47.62, 50.23)	43.67 (42.26, 45.08)	57.26 (56.12, 58.41)	<0.001
20-34	24.10 (21.29, 27.15)	35.23 (31.29, 39.37)	6.45 (4.96, 8.35)	
35-49	24.42 (21.61, 27.46)	27.67 (24.48, 31.10)	19.27 (15.84, 23.23)	
50-64	31.54 (29.07, 34.13)	25.63 (22.89, 28.57)	40.92 (36.96, 45.01)	
65-79	19.94 (17.03, 23.22)	11.48 (9.17, 14.28)	33.36 (28.71, 38.35)	
Gender				0.347
Male	50.21 (47.79, 52.62)	49.13 (45.49, 52.78)	51.92 (47.79, 56.02)	
Female	49.79 (47.38, 52.21)	50.87 (47.22, 54.51)	48.08 (43.98, 52.21)	
Race				0.430
Mexican American	8.52 (6.31, 11.40)	8.98 (6.47, 12.34)	7.78 (5.87, 10.24)	
Other Hispanic	6.84 (5.61, 8.32)	7.19 (5.80, 8.87)	6.29 (4.98, 7.92)	
Non-Hispanic white	63.95 (59.76, 67.94)	62.99 (58.74, 67.05)	65.47 (60.43, 70.19)	
Non-Hispanic black	10.77 (8.31, 13.85)	10.86 (8.28, 14.12)	10.63 (8.00, 14.01)	
Other race	9.92 (8.03, 12.20)	9.98 (7.95, 12.47)	9.82 (7.56, 12.67)	
Education level				<0.001
Less than high school	9.70 (8.43, 11.14)	8.27 (6.87, 9.93)	11.97 (10.31, 13.85)	
High school	26.63 (24.32, 29.08)	23.70 (20.49, 27.25)	31.27 (28.34, 34.36)	
More than high school	63.67 (60.67, 66.57)	68.03 (63.82, 71.96)	56.76 (53.58, 59.89)	
Marital status				<0.001
Married/Living with the partner	63.61 (58.76, 68.19)	62.22 (57.75, 66.49)	65.81 (58.91, 72.09)	
Widowed/Divorced/Separated	19.03 (16.28, 22.13)	16.12 (13.76, 18.78)	23.66 (18.98, 29.07)	
Never married	17.36 (14.57, 20.56)	21.67 (18.51, 25.19)	10.53 (7.87, 13.96)	
Smoking status				0.003
No	56.33 (53.76, 58.87)	60.42 (56.75, 63.99)	49.83 (44.55, 55.12)	
Yes	43.67 (41.13, 46.24)	39.58 (36.01, 43.25)	50.17 (44.88, 55.45)	
Alcohol status				0.780
No	6.00 (4.83,7.42)	6.09 (4.73, 7.79)	5.86 (4.49, 7.60)	
Yes	94.00 (92.58, 95.17)	93.91 (92.21, 95.27)	94.14 (92.40, 95.51)	
Vigorous activity				0.065
No	72.62 (69.37, 75.64)	71.18 (67.44, 74.66)	74.89 (70.77, 78.60)	
Yes	27.38 (24.36, 30.63)	28.82 (25.34, 32.56)	25.11 (21.40, 29.23)	
Moderate activity				0.390
No	50.32 (47.22, 53.41)	49.62 (45.82, 53.43)	51.42 (47.76, 55.07)	
Yes	49.68 (46.59, 52.78)	50.38 (46.57, 54.18)	48.58 (44.93, 52.24)	
Central obesity				<0.001
No	38.31 (35.37, 41.33)	53.10 (49.39, 56.79)	14.85 (12.37, 17.73)	
Yes	61.69 (58.67, 64.63)	46.90 (43.21, 50.61)	85.15 (82.27, 87.63)	
Elevated serum triglycerides				<0.001
No	58.23 (56.13, 60.30)	84.54 (81.83, 86.91)	16.53 (14.28, 19.05)	
Yes	41.77 (39.70, 43.87)	15.46 (13.09, 18.17)	83.47 (80.95, 85.72)	
Reduced serum HDL-C				<0.001
No	49.30 (46.70, 51.89)	75.54 (72.97, 77.93)	7.70 (6.07, 9.72)	
Yes	50.70 (48.11, 53.30)	24.46 (22.07, 27.03)	92.30 (90.28, 93.93)	
Hypertension				<0.001
No	51.40 (47.90, 54.88)	72.47 (68.57, 76.06)	18.00 (14.67, 21.89)	
Yes	48.60 (45.12, 52.10)	27.53 (23.94, 31.43)	82.00 (78.11, 85.33)	
Elevated plasma glucose				<0.001
No	42.62 (39.76, 45.54)	58.36 (54.87, 61.77)	17.67 (14.70, 21.10)	
Yes	57.38 (54.46, 60.24)	41.64 (38.23, 45.13)	82.33 (78.90, 85.30)	
Short sleep				<0.001
No	82.86 (80.26, 85.18)	87.83 (84.31, 90.66)	74.98 (70.94, 78.62)	
Yes	17.14 (14.82, 19.74)	12.17 (9.34, 15.69)	25.02 (21.38, 29.06)	
Depression				<0.001
No	91.44 (90.25, 92.49)	94.69 (93.52, 95.66)	86.28 (83.63, 88.56)	
Yes	8.56 (7.51, 9.75)	5.31 (4.34, 6.48)	13.72 (11.44, 16.37)	
Gallstones				<0.001
No	87.96 (85.53, 90.04)	90.54 (87.97, 92.60)	83.89 (80.52, 86.77)	
Yes	12.04 (9.96, 14.47)	9.46 (7.40, 12.03)	16.11 (13.23,19.48)	

For continuous variables: survey-weighted mean (95% CI), P-value was by survey-weighted linear regression (svyglm).

For categorical variables: survey-weighted percentage (95% CI), P-value was by survey-weighted Chi-square test (svytable).

N, sample size; HDL-C, high-density lipoprotein cholesterol.

**Table 2** illustrates the traits of the study subjects suffering from GSD conditions. After applying the sample weights, GSD patients had an average age of 56.36 years, and a prevalence rate of 12.04%. In addition to education level, smoking status, alcohol status, vigorous activity, moderate activity, and sleep status, there were significant differences in baseline characteristics between the two groups (P< 0.05). Individuals with GSD were more likely to be patients with CircS and to have more number CircS components. These individuals were typically female, older, non-Hispanic white, had central obesity, elevated serum triglycerides, reduced serum HDL-C, hypertension, elevated plasma glucose, depression, and were less frequently never married.

**Table 2:** Characteristics of participants by categories of gallstones status, weighted.

Variables	Groups		*P*-value
	Non-gallstones	gallstones	
	(*N*=3660)	(*N*=466)	
Age(years)	47.91 (46.58, 49.24)	56.36 (54.26, 58.46)	<0.001
20-34	26.27 (23.23, 29.55)	8.23 (4.77, 13.86)	
35-49	24.67 (22.15, 27.38)	22.56 (15.55, 31.55)	
50-64	30.69 (28.42, 33.06)	37.77 (31.08, 44.95)	
65-79	18.37 (15.42, 21.74)	31.44 (25.95, 37.50)	
Gender			<0.001
Male	53.45 (51.11, 55.77)	26.54 (21.49, 32.28)	
Female	46.55 (44.23, 48.89)	73.46 (67.72, 78.51)	
Race			0.048
Mexican American	8.65 (6.36, 11.65)	7.57 (5.07, 11.15)	
Other Hispanic	6.78 (5.57, 8.22)	7.30 (4.53, 11.55)	
Non-Hispanic white	63.20 (58.69, 67.50)	69.44 (62.33, 75.73)	
Non-Hispanic black	11.35 (8.66, 14.74)	6.56 (4.77, 8.97)	
Other race	10.03 (8.11, 12.34)	9.14 (5.89, 13.91)	
Education level			0.391
Less than high school	9.78 (8.39, 11.36)	9.13 (6.26, 13.13)	
High school	26.17 (23.60, 28.92)	29.96 (25.12, 35.31)	
More than high school	64.05 (60.68, 67.28)	60.90 (55.78, 65.80)	
Marital status			0.016
Married/Living with the partner	63.41 (58.50, 68.05)	65.05 (57.36, 72.03)	
Widowed/Divorced/Separated	18.48 (15.75, 21.56)	23.08 (17.14, 30.33)	
Never married	18.11 (15.18, 21.47)	11.87 (8.45, 16.43)	
Smoking status			0.274
No	56.82 (54.72, 58.90)	52.71 (44.00, 61.26)	
Yes	43.18 (41.10,45.28)	47.29 (38.74, 56.00)	
Alcohol status			0.466
No	5.86 (4.68, 7.31)	7.00 (4.27, 11.29)	
Yes	94.14 (92.69, 95.32)	93.00 (88.71, 95.73)	
Vigorous activity			0.253
No	72.23 (69.07, 75.18)	75.46 (68.38, 81.38)	
Yes	27.77 (24.82, 30.93)	24.54 (18.62, 31.62)	
Moderate activity			0.797
No	50.21 (46.63, 53.79)	51.10 (45.58, 56.58)	
Yes	49.79 (46.21, 53.37)	48.90 (43.42, 54.42)	
Central obesity			<0.001
No	41.20 (38.03, 44.44)	17.17 (12.60, 22.97)	
Yes	58.80 (55.56, 61.97)	82.83 (77.03, 87.40)	
Elevated serum triglycerides			<0.001
No	59.81 (57.83, 61.77)	46.66 (38.97, 54.51)	
Yes	40.19 (38.23, 42.17)	53.34 (45.49, 61.03)	
Reduced serum HDL-C			<0.001
No	50.98 (48.48, 53.47)	37.02 (30.04, 44.59)	
Yes	49.02 (46.53, 51.52)	62.98 (55.41, 69.96)	
Hypertension			
No	53.29 (49.84, 56.71)	37.61 (30.10, 45.77)	<0.001
Yes	46.71 (43.29, 50.16)	62.39 (54.23, 69.90)	
Elevated plasma glucose			0.001
No	43.99 (40.62, 47.41)	32.68 (27.54, 38.27)	
Yes	56.01 (52.59, 59.38)	67.32 (61.73, 72.46)	
Short sleep			0.290
No	83.13 (80.61, 85.39)	80.88 (75.17, 85.54)	
Yes	16.87 (14.61, 19.39)	19.12 (14.46, 24.83)	
Depression			<0.001
No	92.29 (91.23, 93.23)	85.19 (80.53, 88.89)	
Yes	7.71 (6.77, 8.77)	14.81 (11.11, 19.47)	
Circadian syndrome			<0.001
No	63.11 (60.76, 65.41)	48.22 (41.34, 55.17)	
Yes	36.89 (34.59, 39.24)	51.78 (44.83, 58.66)	
Components of circadian syndrome			<0.001
≤3	63.11 (60.76, 65.41)	48.22 (41.34, 55.17)	
4	19.25 (17.58, 21.04)	18.10 (13.51, 23.80)	
5	13.88 (12.08, 15.90)	22.68 (18.42, 27.59)	
≥6	3.76 (3.25, 4.33)	11.00 (7.08, 16.70)	

For continuous variables: survey-weighted mean (95% CI), P-value was by survey-weighted linear regression (svyglm).

For categorical variables: survey-weighted percentage (95% CI), P-value was by survey-weighted Chi-square test (svytable).

N, sample size; HDL-C, high-density lipoprotein cholesterol.

### Associations between CircS and GSD

**Figure 2:**
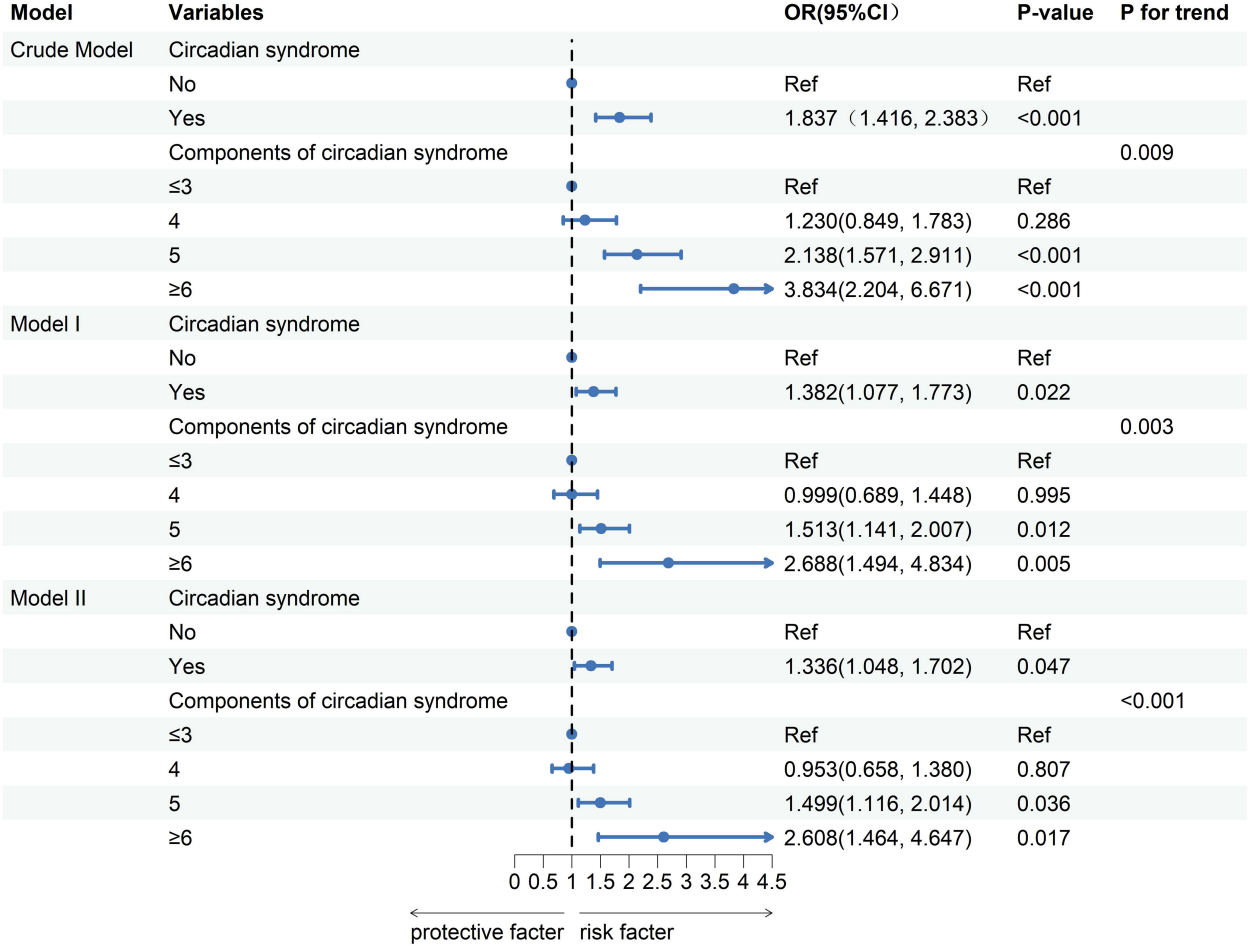
Logistic regression analysis between circadian syndrome and the prevalence of gallstones. OR, odds ratio; CI, confidence interval. Crude Model: unadjusted. Model I: adjusted for age, sex, and race. Model II: adjusted for Model I, education, marital status, smoking status, alcohol consumption, vigorous activity, and moderate activity.

In **Figure 2**, the relationship between Circs and GSD is depicted. Unadjusted for confounding variables, the crude model revealed a positive correlation between Circs and GSD (Odds Ratio (OR) = 1.837, 95% CI: 1.416, 2.383). The same trend was observed in Model I (OR = 1.382, 95% CI: 1.077, 1.773) and Model II (OR = 1.336, 95% CI: 1.048, 1.702), suggesting that there remained a positive correlation between CircS and GSD even after accounting for potential confounders. When the data were stratified by the number of Circs components, a positive correlation was observed between the number of CircS components and the prevalence of GSD (P for trend < 0.05). In particular, individuals with six or more CircS components had a higher prevalence of GSD than those with three or fewer components (OR = 2.608, 95% CI: 1.464, 4.647).

### Subgroup analysis

**Figure 3:**
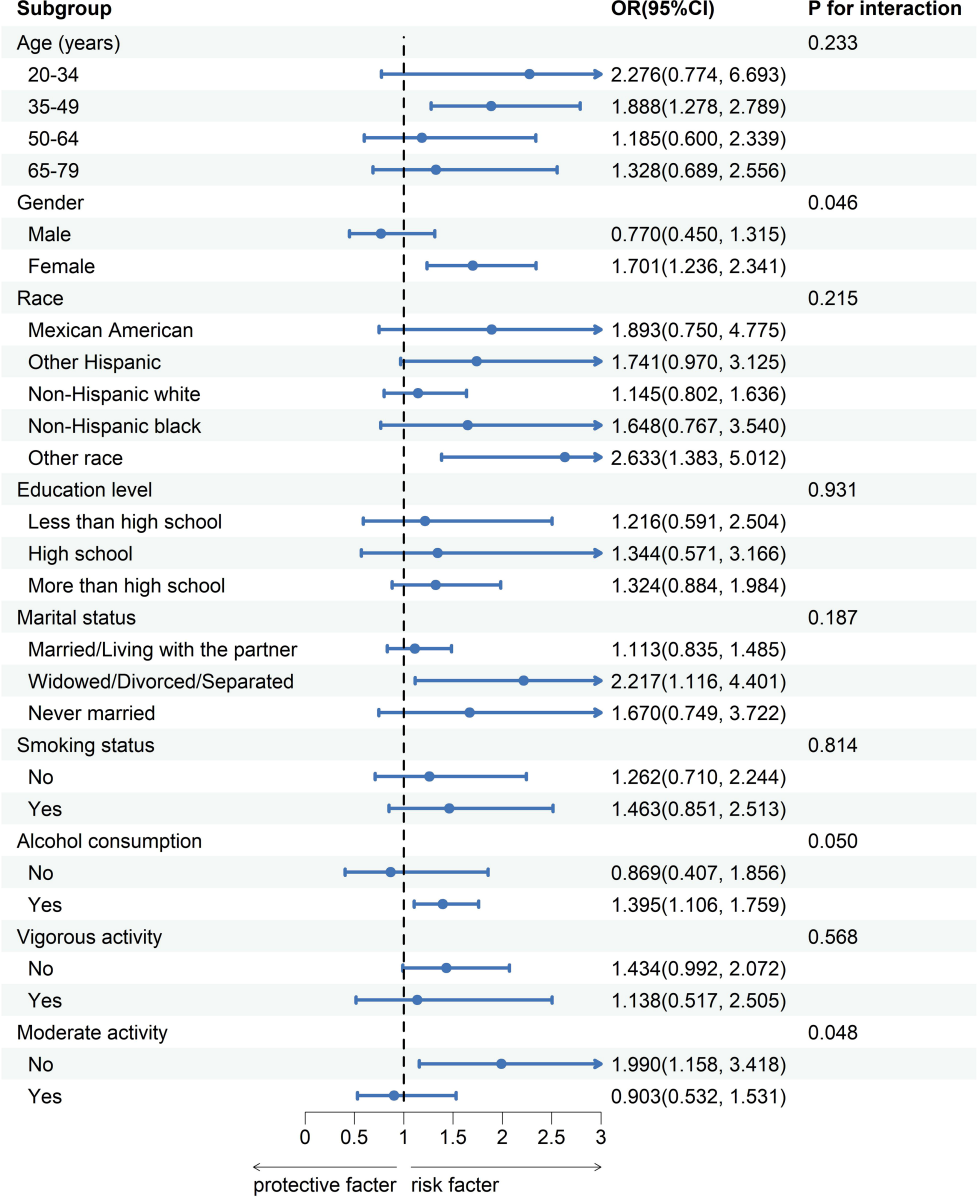
Association between circadian syndrome and the incidence of gallstones according to subgroup and interaction analyses. OR, odds ratio; CI, confidence interval. Multivariable logistic regression models were adjusted for age, sex, race, education, marital status, smoking status, alcohol consumption, vigorous activity, and moderate activity. Stratification variables were not adjusted in the corresponding models.

To evaluate the uniformity of the correlation between Circs and GSD among the general individuals, we performed the subgroup analysis and interaction test considering factors such as age, gender, ethnicity, educational background, marital status, smoking habits, alcohol status, vigorous activity, and moderate activity. The findings revealed a non-uniform correlation between Circs and GSD among various subgroups. As shown in **Figure 3**, the correlation between Circs and GSD interacted with gender and moderate activity (P for interaction < 0.05). In female individuals (OR=1.701, 95%CI: 1.236, 2.341) or those not engaged in moderate activity (OR=1.990, 95%CI: 1.158, 3.418), a positive correlation between Circs and GSD was observed. Conversely, In male individuals (OR=0.770, 95% CI: 0.450, 1.315) or those engaged in moderate activity (OR=0.903, 95% CI: 0.532, 1.531), no positive correlation between CircS and GSD was observed. Furthermore, our research revealed an absence of interaction related to age, race, education, marital status, smoking habits, alcohol status, or vigorous activity on the correlation between Circs and GSD (P for interaction > 0.05).

## Discussion

Gallstone disease, a widespread worldwide disease, presents a major risk to both human health and life quality. Recently, with the emergence of the CircS concept, its potential links to a variety of chronic diseases have gradually become a focal point of research. Nonetheless, there is still a limited amount of scientific proof concerning the connection between CircS and GSD. The research utilized a cross-sectional approach, examining NHANES data from 2017 to March 2020 in the U.S., to uncover a positive correlation between CircS and GSD. Remarkably, this association was especially evident in female participants and those who were not involved in moderate activities.

As far as we are aware, this research pioneers the investigation into the connection between CircS and GSD. Earlier studies have predominantly concentrated on the links between GSD and individual factors like MetS, depression, and sleep deprivation. In contrast, this study innovatively incorporates these factors into a unified analytical framework. With the acceleration of modern lifestyles and work rhythms, the incidence of CircS has been increasing annually. CircS encompasses multiple components of MetS, including central obesity, high serum triglyceride levels, decreased serum HDL-C, elevated blood sugar, hypertension, sleep deprivation, and depression, all of which are linked to a heightened risk of GSD. For example, Chen et al. revealed through a cross-sectional study that the more components of MetS are present, the higher the prevalence of GSD (27). Zhu et al. further confirmed through a Mendelian randomization study that MetS can increase the risk of GSD, particularly in MetS patients with abdominal obesity (29). A multicenter cross-sectional study involving 548,934 participants indicated that low levels of HDL-C and high levels of triglycerides are risk factors for GSD (45). Yan et al. confirmed through observational studies and Mendelian randomization that type 2 diabetes can increase the risk of GSD (46). A cohort study in Japan involving 611,930 participants showed that hypertension can increase the risk of GSD (47). He et al. disrupted the circadian rhythm in adult male C57BL/6J mice by feeding them lithogenic diets during their sleep phase and found that circadian rhythm disruption can increase the risk of GSD in mice (31). Li et al. through retrospective cohort studies and mediated Mendelian randomization research, found that patients with depression have a higher risk of GSD and revealed that abdominal obesity plays a mediating role (32). These findings suggest a close association between CircS and the risk of GSD. Moreover, the primary cause of CircS is believed to be a malfunction in circadian rhythms. A multitude of elements, such as inconsistent sleep habits, regular night shifts, and extended exposure to artificial light, along with genetic influences or specific health issues, may result in a discrepancy between the internal circadian rhythm and the external environment’s 24-hour cycle, leading to disorders of the circadian rhythm (48). The onset of circadian rhythm issues can lead to various physiological and pathological states, such as MetS, type 2 diabetes, depression, and heart-related illnesses (49–51). A cohort study spanning from 2010 to 2018, which collected work hour data from 5,775 hospital staff in Taiwan, revealed that night shift work, compared to day shift work, may increase the risk of MetS and abdominal obesity. The study also found that among night shift workers, the frequency of night shifts is associated with an increased risk of hypertension (52). Furthermore, a meta-analysis confirmed a significant association between night shift work and the risk of MetS, showing that this risk is positively correlated with the duration of night shift work (53). Additionally, several studies have indicated that shift work may not only increase the risk of type 2 diabetes, but also the risk of myocardial infarction, cerebrovascular accidents, coronary artery disease, and reperfusion injury after myocardial infarction (54–57). The existence of these diseases could play a role in the development of gallstones. This study’s findings align with expectations, showing a positive link between CircS and the likelihood of developing GSD. Following CircS criteria, which necessitate a minimum of four traits, we further explored whether the quantity of these traits correlates with the likelihood of gallstones. The findings are quite intriguing: a notable positive link exists between the quantity of CircS traits and the probability of gallstone formation. In other words, an increased number of CircS characteristics in a person correlates with a higher likelihood of gallstone formation.

The link between CircS and GSD prevalence may be mediated through several biological mechanisms. First, the liver, a key organ in the regulation of circadian rhythms, may experience rhythmic disruptions that can lead to an imbalance in bile acid metabolism (58, 59). Bile acids play a crucial role in dissolving cholesterol in bile, maintaining cholesterol balance, and preventing excessive cholesterol accumulation. A disruption in the metabolism of bile acids is deemed the primary pathological factor in the development of gallstones (60, 61). Consequently, CircS may facilitate gallstone development by interfering with the metabolic pathways of bile acids. Additionally, adiponectin, a protein hormone that plays a role in controlling glucose and fatty acid metabolism, has emerged as a central research subject in recent years. It has been established that adiponectin and the circadian rhythm interact bidirectionally (62). A lack of sleep may result in reduced levels of adiponectin in the blood, and lacking adiponectin may encourage the development of gallstones in mice (63, 64). This suggests that CircS may facilitate the onset and progression of gallstones by reducing adiponectin levels. Third, the circadian clock, as an intricate cellular mechanism, not only regulates various physiological processes but also controls inflammatory responses (65). The circadian clock can directly interact with the key elements of the critical inflammatory pathway. Likewise, inflammation may result in disruptions of circadian rhythms, potentially intensifying the inflammatory reaction and exacerbating tissue damage (66). Significantly, inflammatory reactions play a crucial role in gallstone development, where heightened proinflammatory factor levels are closely linked to a greater likelihood of gallstones (67). Therefore, inflammatory responses may play an important intermediary role between CircS and the prevalence of gallstones. Finally, the link between CircS and the prevalence of gallstones may also involve the homeostasis of the gut microbiota. There is an undeniable connection between circadian rhythms and the balance of the gut microbiota (68). Studies show that lack of sleep significantly diminishes gut microbiota diversity, which in turn lowers short-chain fatty acid production, exacerbates intestinal inflammation, and heightens intestinal permeability (69). These disruptions in the gut microbiota balance may, through a series of complex physiological mechanisms, promote the formation and progression of gallstones (70, 71).

Furthermore, this study revealed differences in the correlation between CircS and GSD in terms of gender and moderate activity. The results indicate that this connection is particularly evident in female CircS patients. This occurrence could be intimately linked to disparities in hormone levels and psychosocial elements between genders. The hormonal fluctuations accompanying the menstrual cycle and menopause in women, especially the cyclical changes in estrogen and progesterone, have been shown to affect bile composition and gallbladder function. These hormone fluctuations may lead to cholesterol supersaturation in bile, thereby increasing the likelihood of cholesterol crystal and stone formation (72, 73). Additionally, women may be more susceptible to the influence of stress and emotional issues, which can further affect bile metabolism and gallbladder function through their effects on the autonomic nervous system and hormone levels (74). The presence of circadian rhythm disturbances may exacerbate hormonal fluctuations and the influence of psychosocial factors, thereby increasing the risk of GSD in female CircS patients (75–77). Furthermore, the study also found that the positive correlation between CircS and GSD was significant in individuals with CircS who were not engaging in moderate activity. Moderate activity plays a beneficial role in maintaining metabolic balance and promoting bile flow. Moderate-intensity activity is widely believed to improve various components of metabolic syndrome, including reducing blood pressure, improving blood sugar, reducing abdominal fat accumulation, and increasing HDL-D levels, thus helping to reduce cholesterol supersaturation and lowering the risk of gallstone formation (78). Physical activity also promotes gallbladder contraction, improves bile flow, helps prevent bile stasis and cholesterol crystallization, and reduces the formation of gallstones. Individuals not engaging in moderate activity may experience decreased gallbladder activity and slowed bile flow, leading to cholesterol deposition and stone formation in the gallbladder (79). Additionally, psychosocial factors may also play an important role in the relationship between moderate activity and the incidence of cholesterol stones. Physical activity has been shown to reduce stress and anxiety and improve emotional states (80). Individuals not engaging in moderate activity may be more susceptible to stress and emotional issues, increasing the risk of gallstone formation. Therefore, this study emphasizes the potential importance of moderate activity in preventing the occurrence of GSD in patients with CircS, suggesting that we should actively advocate for patients with CircS, especially women and those who do not regularly engage in physical activity, to participate in moderate-intensity activity regularly to reduce the risk of gallstone disease and improve quality of life.

This study has significant strengths. First, the research is underpinned by the NHANES database, which offers nationwide coverage, enhancing the representativeness and generalizability of our findings. Second, we introduce the concept of CircS, a novel construct based on the understanding of the human internal biological clock or circadian rhythm, and explore its potential link with GSD. Nonetheless, this research has its own set of constraints. The NHANES database is cross-sectional, providing data from a single time point without longitudinal follow-up information, which limits our ability to delve into the causal relationships and mechanisms between CircS and GSD. Additionally, all diagnoses of gallstones in the study were self-reported, which may lack the precision of diagnoses confirmed by medical imaging. Furthermore, although we endeavored to control for known risk factors in our analysis to investigate the relationship between these two conditions, we must acknowledge that there may be unidentified factors that could affect or obscure this relationship. Nonetheless, this study lays a solid foundation for broader and more in-depth prospective research in the future.

## Conclusions

This study suggests that CircS is associated with an increased risk of GSD prevalence, and the more components of CircS, the higher the risk of GSD prevalence. This association is particularly evident among females and individuals who are not engaging in moderate activity. These findings not only provide new perspectives for future research directions in GSD but also have significant implications and carry substantial consequences for crafting strategies for prevention and treatment. However, establishing a causal relationship between CircS and gallstones requires further investigation through additional high-quality prospective cohort studies.

## Data Availability

Publicly available datasets were analyzed in this study. This data can be found here: https://www.cdc.gov/nchs/nhanes/index.htm.
